# Policy lessons from physicians’ strikes

**DOI:** 10.1186/2045-4015-2-34

**Published:** 2013-09-17

**Authors:** Gregory P Marchildon

**Affiliations:** 1 Johnson-Shoyama Graduate School of Public Policy, University of Regina, 110 – 2 Research Drive, S4S 0A2 Regina, Saskatchewan, Canada

## Abstract

Drawing upon the literature on physicians’ strikes from other OECD countries, the experience with physician strikes in Israel is put into comparative perspective. There are both structural and ideological factors that help to explain why there have been more strikes in Israel relative to other countries. At the same time, the dynamics of the strike and divisions within the medical profession in Israel, may be contributing to policy drift. This is a commentary on http://www.ijhpr.org/content/2/1/33.

## 

In their article, Leonora Weil, Gabi Bin Nun and Martin McKee analyse the reasons for a series of doctors’ strikes and work actions in Israel between April and November 2011, the first breakdown between the government and doctors following the ten-year agreement originally concluded in 2000. Although not involving all doctors, this was the longest running industrial action by physicians in Israel’s history, a history pockmarked by physician strikes from 1973 until 2000. The question asked by the authors is whether the more recent series of work actions was solely a reflection of physician unhappiness with remuneration and working conditions or a more profound symptom of a crisis in the publicly funded health system [1].

This article is a welcome contribution to the sparse literature on physicians’ strikes. Weil, Nun and McKee note that there is almost no scholarly literature on physicians’ strikes in Israel. The situation is not much better outside Israel despite the fact that physicians who strike against the state pose a major threat to the government in power, and bring into question the state’s stewardship of public health care. However, unlike the case of Israel, physician strikes appear to have been quite rare in the wealthier OECD countries concentrated in Western Europe, Australasia and North America [2]^a^. I would suggest that this has been, and continues to be, the case for both structural and ideological reasons.

In the majority of wealthy OECD countries, physicians are in fact independent professionals whose contracts with the state as well as payment mechanisms give them a level of independence much greater than other health workers. Their interests are powerfully represented by organized medicine which has done its utmost to protect their professional autonomy and income. In his 1991 comparative review of the power of organized medicine, David Mechanic found little evidence supporting the conventional wisdom that the medical profession’s influence had declined since the Second World War [3].

Indeed, it would only make logical sense for organized medicine to become more involved in protecting the status and position of their members as government became increasingly involved in the funding, regulating and administrating of health care. In a number of the richer OECD countries, the majority of physicians initially opposed the establishment of universal coverage. (However, it should be noted, once it was clear that more public coverage was inevitable, organized medicine worked with the state on the program details to obtain maximum independence and income for their members.) Based on a study that analyzed physician strikes in Canada and Belgium, this opposition was in part the consequence of the majority of doctors sharing a common ideology – what we termed medical liberalism [4].

At the heart of medical liberalism is the assumption that physicians are, or should be, independent, self-employed actors with the freedom to determine the nature of their relationship with patients and professional peers, as well as freedom from state interference in their clinical decisions and judgments. In those countries with professional self-regulation, this freedom includes physician control over medical education, standards and ethics. The self-employed ethos of physicians in these countries means that they are more likely to view strike action as incompatible with the professional and ethical responsibilities, and they are less likely to identify with other health workers (e.g. nurses) who have more frequently relied on strikes to obtain their goals [5]^b^.

So why, given this, have there been more physician strikes in Israel than in the wealthier OECD countries? I think there may be some important structural differences. Compared to their counterparts in most (and there are a few exceptions^c^) of the richer OECD countries, a larger percentage of Israeli physicians are directly employed on salary by the state or state-funded organizations. While some supplement their income by doing private work – usually on a fee-for-service basis – this means that the relationship between the “average” physician and the state is much closer than in the majority of wealthy OECD countries where physicians remain independent contractors even if they work in public hospitals and clinics. This means that the government in Israel has greater control over both remuneration and working conditions.

Salaried physicians within hierarchical organizations are less likely to draw a sharp distinction between themselves and other health workers and are more prone to see a strike as a justifiable means to improve their income and working conditions. As in the Israeli case, this may be layered on by more social objectives, including facilitating improved access to medical care in peripheral regions by providing additional incentives for doctors to work in such regions.

Physicians do appear to be more ideologically divided in Israel than the OECD countries I have covered. The physicians who launched the 2011 strike expressed significant dissatisfaction about the trajectory of public health care in Israel and genuine concern about long-standing access issues, based on the consistently declining public share since universal coverage was introduced in 1995. In contrast, other factions – including the younger doctors who mobilized in the second wave of the 2011 strike – appear to be more concerned about improving their income and increasing their independence through additional, non-governmental, sources of income. In particular, many of them want the state to permit dual practice – public patients in the morning and private patients later in the day – irrespective of the negative policy consequences for the public system. I am not sure, but I would guess that this second group of physicians enthusiastically accepts the main tenets of medical liberalism.

If there are sharp divisions in the medical profession, then a strike can actually exacerbate these differences as any gain by one faction are potentially viewed as a loss by the others. It can also facilitate policy drift by the government in power; that is, allowing it to politically steer a given policy (e.g. universal health care coverage) away from its original scope and purpose even while making the result appear a natural consequence of factors beyond the government’s control [6].

Moreover, faced by incompatible demands from a divided medical profession, the government can minimize political and fiscal risk by lowballing the final settlement – conceding the minimum possible in terms of the profession’s original demands. This allows the government to avoid hard directional decisions on policy in terms of changing the structures of the original policies even while subverting their original purpose. For example, while the government might pay doctors more to provide additional access in peripheral regions, it is unwilling to reverse the quiet but steady decline in the public share of funding. At the same time, the government is not prepared to reverse the rules prohibiting dual practice as it does not want to change the structures of the original policy in order to avoid being seen as actively undermining the public health system.

## Endnotes

^a^However, this must remain a tentative statement until a systematic and comprehensive comparison of physicians’ strikes across countries is completed.

^b^In several OECD countries, the government's is involved in setting physician fee schedules. Further research is needed to explore the extent to which this provides an impetus for work actions and why this is so.

^c^Based on a study of 14 OECD countries, in only two (Iceland and Finland) were the general practitioners (GPs) salaried as opposed to self-employed, while four countries beyond Iceland and Finland (United Kingdom, Denmark, Hungary and the Czech Republic) had salaried rather than self-employed specialists [7]. Further research would be needed to understand why this has not led to more strike activity in the minority of countries with salaried physicians.

## Competing interests

The author declares that he has no competing interests.

## Authors’ information

GPM is currently Canada Research Chair and Professor at the Johnson-Shoyama Graduate School of Public Policy, University of Regina, and a fellow of the Canadian Academy of Health Sciences. Previously, he served as the Executive Director of the Commission on the Future of Health Care in Canada and was a faculty member at Johns Hopkins University’s School of Advanced International Studies.

## Commentary on

Weil LG, Nun GB, McKee M: Recent physician strike in Israel: a health system under stress? Isr J Health Policy Res 2013, 2:33.
